# Performance of the CORB (Confusion, Oxygenation, Respiratory Rate, and Blood Pressure) Scale for the Prediction of Clinical Outcomes in Pneumonia

**DOI:** 10.1155/2022/4493777

**Published:** 2022-06-03

**Authors:** Luis F. Reyes, Alirio R. Bastidas, Eduardo Tuta Quintero, Juan S. Frías, Álvaro F. Aguilar, Karen D. Pedreros, Manuela Herrera, Laura D. Saza, Alejandra P. Nonzoque, Laura E. Bello, Maria D. Hernández, Germán A. Carmona, Anyelinne Jaimes, Silvia M Ramírez, Natalia Murillo

**Affiliations:** ^1^Universidad de La Sabana, Chía, Cundinamarca, Colombia; ^2^Clínica Universidad de La Sabana, Chía, Cundinamarca, Colombia

## Abstract

**Background:**

Community-acquired pneumonia (CAP) is a common cause of morbidity and mortality due to misdiagnosis and inappropriate treatment approaches.

**Objective:**

To assess the performance of the CORB score in subjects with CAP for predicting in-hospital mortality, death within 30 days of admission, and requirement for invasive mechanical ventilation (IMV) and vasopressor support.

**Methods:**

A retrospective, cohort study with diagnostic test analysis of CORB and CURB-65 scores in subjects with CAP according to ATS criteria was undertaken. An alternative CORB score was estimated by replacing SpO_2_ ≤90% by the SpO_2_/FiO_2_ ratio. Crude and adjusted odd ratios (AOR) were calculated for each variable. The area under the receiver operating characteristics curve (AUROC) was constructed for each score, and outcomes were analyzed. AUROCs were compared with the DeLong test, considering a *p* value <0,05 statistically significant.

**Results:**

From 1,811 subjects who entered the analysis, 15.1% (273/1,811) died in hospital, 8.78% required IMV (159/1,811), and 9.77% (177/1,811) needed vasopressor support. CORB had an AUROC of 0,660 (95% CI: 0,623–0,697) for in-hospital mortality; an AUROC of 0,657 (95% CI: 0,621–0,692) for 30-day mortality; an AUROC of 0,637 (CI 95%: 0,589–0,685) for IMV requirement; and an AUROC of 0,635 (95% CI: 0,589–0,681) for vasopressor support. CORB performance increases when the SpO_2_/FiO_2_ ratio <300 is used as oxygenation criterion in the prediction of requirement for IMV and vasopressor support, with AUROC of 0,700 (95% CI: 0,654–0,746; *p* < 0.001) and AUROC of 0,702 (95% CI: 0,66–0,745; *p* < 0.001), respectively. CURB-65 score presents an in-hospital mortality AUROC of 0,727 (95% CI: 0,695–0,759) and 30-day mortality AUROC of 0,726 (95% CI: 0,695–0,756).

**Conclusions:**

CORB score has a good performance in predicting the need for IMV and vasopressor support in CAP patients. This performance improves when the SpO_2_/FiO_2_ ratio <300 is used instead of the SpO2 ≤90% as the oxygenation parameter. CURB-65 score is superior in the prediction of mortality.

## 1. Introduction

Community-acquired pneumonia (CAP) continues to be one of the main causes of morbidity and mortality; its occurrence in adults is estimated at approximately 16 to 23 cases per 1,000 person-years, and it increases with age [1]. It is the leading cause of death from infection worldwide [2, 3]. A global mortality of 10% to 14% is attributed to it, being less than 2% in healthy young people. However, mortality can increase to 11%–14% in adults who require hospitalization and may reach 25% to 50% in patients who are admitted to an intensive care unit (ICU) [4, 5]. Its early recognition and treatment are essential to avoid an ICU delayed admission, which is considered an independent factor related to a long hospital stay and higher mortality rates [6]. For this reason, different scores have been created and validated to carry out an effective prognostic identification, thus providing a guide to the most appropriate site for handling and monitoring the CAP patient.

Among the most widely used scores to predict mortality are the PSI (pneumonia severity index) [5, 7] and CURB-65 (confusion, urea nitrogen >7 mmol/L (19 mg/dL), respiratory rate ≥30/min, systolic blood pressure <90 mmHg or diastolic blood pressure ≤60 mmHg, and age ≥65 years) [8]. Additionally, given their superiority in predicting the need for ICU or mechanical ventilation, the criteria for pneumonia severity of the Infectious Diseases Society of America/American Thoracic Society (IDSA/ATS) [5, 8] and the SMART-COP score have been used [9]. However, one of the restrictions to applying these scores in different care settings is the need to perform at least one invasive procedure.

In search of a practical score that does not require the use of invasive measures in its construction, Buising et al. [10] proposed in 2007 the CORB score, which uses the information of consciousness state, oxygen saturation by pulse oximetry, respiratory rate, and blood pressure, reaching a sensitivity of 72.2% and a specificity of 70.1% for a composite outcome of mortality and requirement for invasive mechanical ventilation (IMV) and vasopressor support [10, 11]. Nevertheless, performance data of CORB score are still scarce for independent prediction of these outcomes, and its performance related to scores such as the CURB-65 is not clear, which is why this study proposes to assess performance of the CORB score compared to the CURB-65 score as a predictor of IMV, vasopressor support, in-hospital mortality, and 30-day mortality.

## 2. Material and Methods

A retrospective cohort study was carried out in subjects with CAP in the third-level center Clínica Universidad de La Sabana (Chía, Cundinamarca, Colombia). Patients were treated in the emergency department, general ward, or ICUs. Data were collected from January to August 2020, from medical records dated between January 2012 and February 2020.

### 2.1. Selection Criteria

Inclusion criteria included an age ≥18 years, regardless of the gender; stay in the emergency room (resuscitation, observation room, and transit room), general ward, or ICU (for at least 6 hours); CAP diagnosis according to the ATS guidelines [5, 7]; and clinical records that included information for CORB and CURB-65 scores' assessment. Patients with acute decompensation of chronic diseases such as exacerbated COPD, previous congenital heart disease or decompensated heart failure, and chronic or acute interstitial lung disease were excluded. Patients whose pneumonia diagnosis changed or was ruled out during hospitalization, whose pneumonia was related to bronchial obstruction, or those who required IMV prior to taking arterial gases were also excluded.

### 2.2. Variables

The requirement for IMV and vasopressor support, in-hospital death, and 30-day mortality were the outcomes; the composite outcome included the variables of in-hospital mortality, IMV requirement, and vasopressor requirement. In addition, clinical presentation, findings on physical examination, vital signs, FiO_2_ and SpO_2_ upon admission, comorbidities, laboratory tests results, diagnostic imaging findings (chest X-ray and/or chest CT), and arterial blood gases were considered as independent variables.

With the obtained variables, CURB-65 and CORB were calculated; the latter scores one point for the variables confusion, oxygenation by SpO_2_ ≤90%, respiratory rate ≥30 breaths/minute, and blood pressure (systolic blood pressure <90 mmHg or diastolic blood pressure ≤60 mmHg), with a score of ≥2 points being considered as severe pneumonia. In addition, a CORB score was constructed in which the oxygenation assessment of SpO_2_ ≤90% was replaced by a SpO_2_/FiO_2_ ratio <300.

Data were obtained from admission registry and clinical records during entire hospital stay, while 30-day mortality was obtained from the national source of death. In order to reduce transcription biases, medical records were revised by at least two different reviewers, and data were verified by them when they were recorded into the database.

### 2.3. Sample Size

Sample size estimation in diagnostic test [11] and data from the study by Williams et al. [12] were considered, in which an incidence of in-hospital mortality due to CAP of 12.7% and a performance for this outcome of the CORB score ≥2 points of 78% for sensitivity and 40% for specificity were reported. For a confidence level of 95% and a precision level of 7%, a minimum total of 1,060 subjects were required. Patients who did not meet the eligibility criteria were replaced until the sample size was completed.

### 2.4. Statistical Analysis

Data were compiled in the electronic data capture software Research Electronic Data Capture (REDCap) [13] and later downloaded to a spreadsheet to perform the final analysis in the licensed in STATA 14 and SPSS 25 program. Qualitative variables were reported in frequencies and percentages, while quantitative variables were summarized in median and interquartile ranges if their distribution was normal in mean and standard deviation and if their distribution did not meet normality parameters. A bivariate analysis was performed comparing the quantitative variables with Student's *t* or Mann–Whitney U test according to their distribution, whereas the qualitative variables were compared using the Chi-square test. By using the scores obtained from CURB-65 and two CORB scores (one score with SpO_2_ and another one with the SpO_2_/FiO_2_ ratio as oxygenation parameters), the respective areas under the ROC curve (AUC-ROC), sensitivity, specificity, positive predictive value (PPV), negative predictive value (NPV), positive likelihood ratio (LR+), and negative likelihood ratio (LR−) with their respective 95% confidence intervals were determined. The association strength of each variable of the studied scores regarding the proposed outcomes was estimated by calculating the odds ratio (OR) and adjusted odds ratio (AOR) using a logistic regression model. The ACORs of the different scores were compared with the DeLong test. For the estimates, a *p* value <0,05 was considered significant.

### 2.5. Ethical Considerations

This study was approved by the research committee of the Universidad de La Sabana and the institutional ethics committee of the Clínica Universidad de La Sabana, as registered on Act Minute No 526 of January 29, 2021.

## 3. Results

**Figure 1:**
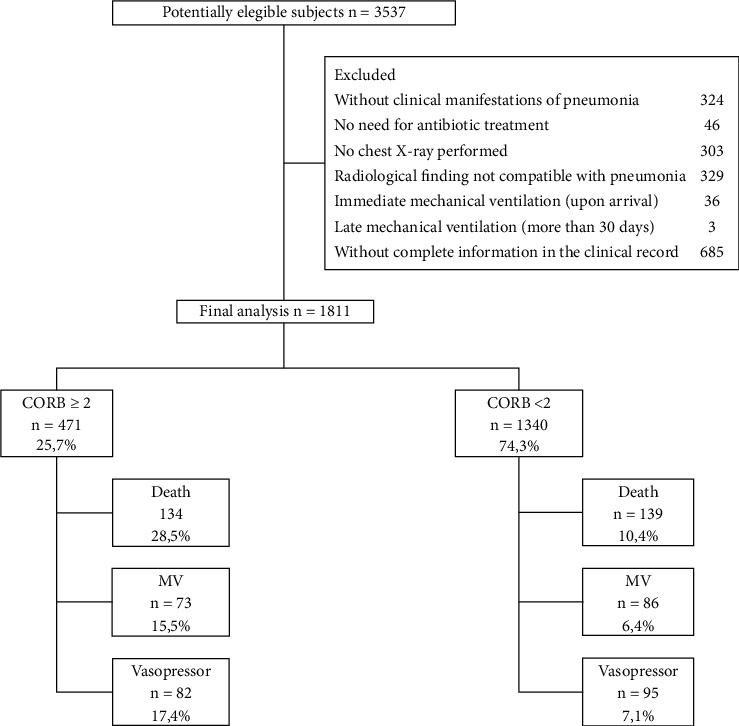
Flowchart of study subjects' entry. FiO2: fraction of inspired oxygen. CORB: confusion (new onset or deterioration of preexisting condition), oxygen saturation ≤90%, respiratory rate ≥30/min, and systolic blood pressure <90 mmHg or diastolic blood pressure ≤60 mmHg. BUN: blood urea nitrogen. IVM: invasive mechanical ventilation requirement. ICU: intensive care unit.

1,811 subjects were admitted to the final analysis; 15.1% (273/1,811) died in hospital care, 8.78% (159/1,811) required IMV, and 9.77% (177/1,811) required vasopressor support. 25.7% (471/1,811) subjects had a CORB score ≥2 points; the flow of study subjects' entry is described in Figure 1.

### 3.1. Population Characteristics

The mean age of the patients was 72.89 years (SD ± 17.09); 56.6% (1,025/1,811) were male. The most frequent symptom was cough in 85.8% (1,554/1,811). The most prevalent comorbidity was high blood pressure in 63.1% (1,142/1,811), and multi-lobar involvement was observed in 30.1% (545/1,811) of the patients. Significant relationship was found for mortality outcome with age: 80.3 vs. 71.6 years (*p* < 0.001); presence of dyspnea: 82.4% (225/273) vs. 75.8% (1,166/1,538) (*p* = 0.017); presence of rales: 72.5% (198/273) vs. 64.5% (992/1,538) (*p* = 0,010); presence of retractions: 54.6% (149/273) vs. 30.6% (471/1,538) (*p* < 0.001); history of high blood pressure: 72.5% (198/273) vs. 61.4% (944/1,538) (*p* < 0.001); chronic heart failure: 31.5% (86/273) vs. 20% (308/1,538) (*p* < 0.001); and dementia: 34.8% (95/273) vs. 13.6% (209/1,538) (*p* < 0.001).  Table 1 summarizes other general characteristics of the population.

Calculated odds ratios for each variable belonging to the CORB and CURB-65 scores were different and dependent on whether the assessed outcome was mortality, requirement for IMV, or requirement for vasopressor support. In the case of in-hospital mortality, the OR for confusion was 4.1 (95% CI: 3,114–5,527; *p* < 0.001) and when 30-day mortality was evaluated, the OR was 1,4 (95% CI: 1,262–1,505; *p* < 0.001); the OR for IMV was 3,1 (95% CI: 2,142–4,348; *p* < 0.001) and for vasopressor support was 2,8 (95% CI: 2–3,967; *p* < 0.001).

The variables with the highest OR for mortality outcomes were altered state of consciousness and impaired oxygenation with SpO_2_/FiO_2_ <300 (confusion OR = 4.1; 95% CI: 3,114–5,527; *p* < 0.001 for in-hospital mortality. OR = 1.4; 95% CI: 1,262–1,505; *p* < 0.001 for 30-day mortality. SpO_2_/FiO_2_ <300 OR = 4.2; 95% CI: 3,136–5,622; *p* < 0.001 for in-hospital mortality. OR = 1.4; 95% CI: 1,249–1,496; *p* < 0.001 for 30-day mortality). Variables with the highest OR for the outcome of IMV and vasopressor support were RR ≥ 30 rpm and oxygenation by SpO_2_/FiO_2_ <300. The OR and adjusted OR for each of the different score variables and outcomes of interest are shown in Supplementary File 1.

The dichotomous scoring on the CURB-65 and CORB scales was statistically significant for all outcomes; however, in the IMV and vasopressor support requirement outcomes, when SpO_2_ ≤ 90% is replaced by SpO_2_/FiO_2_ <300 in the CORB score, the OR is higher (OR of 2,7 and OR of 2,8 with CORB and SpO_2_ ≤ 90% for IMV and vasopressor support vs. OR of 5,0 and OR of 4,3 with CORB and SpO_2_/FiO2 <300, respectively). The OR for composite outcomes of CURB-65, CORB >2, and CORB >2 (SpO_2_/FiO2 < 300) is shown in Table 2.

### 3.2. CORB and CURB-65 Scores' Performance for Mortality, IMV Requirement, and Vasopressor Support Requirement

The AUC-ROC of the CURB-65 score for in-hospital and 30-day mortality was 0,727 (95% CI: 0,695–0,759; *p* < 0.001) and 0,726 (95% CI: 0,695–0,756; *p* < 0.001), respectively, while the AUC-ROC of the CORB score with SpO_2_ ≤90% for in-hospital and 30-day mortality was 0,660 (95% CI: 0,623–0,697; *p* < 0.001) and 0,657 (95% CI: 0,621–0,692; *p* < 0.001), with DeLong test *p* < 0.001.

For the outcomes of vasopressor support and IMV, the CURB-65 showed AUC-ROC of 0,608 (95% CI: 0,562–0,654; *p* < 0.001) and 0,587 (95% CI: 0,538–0,637; *p* < 0.001) respectively, while the CORB showed an AUC-ROC of 0,635 (95% CI: 0,589–0,681; *p* < 0.001) for vasopressor support and an AUC-ROC of 0,637 (95% CI: 0,589–0,685; *p* < 0.001) for IMV, with DeLong test *p* < 0.001. When the SpO_2_ ≤90% is replaced by SpO_2_/FiO_2_ <300 in the CORB score, the AUC-ROC improves for predicting the requirement for vasopressor support and IMV, with AUC-ROC of 0,700 (95% CI: 0,654–0,746; *p* < 0.001) and AUC-ROC of 0,702 (95% CI: 0,66–0,745; *p* < 0.001), respectively. The AUC-ROC of the CORB score that uses SpO_2_/FiO_2_ <300 exceeds the CURB-65 score by 0,09 and 0,11 points for the outcomes of vasopressor support and IMV, respectively, with DeLong test *p* < 0.001.

**Figure 2:**
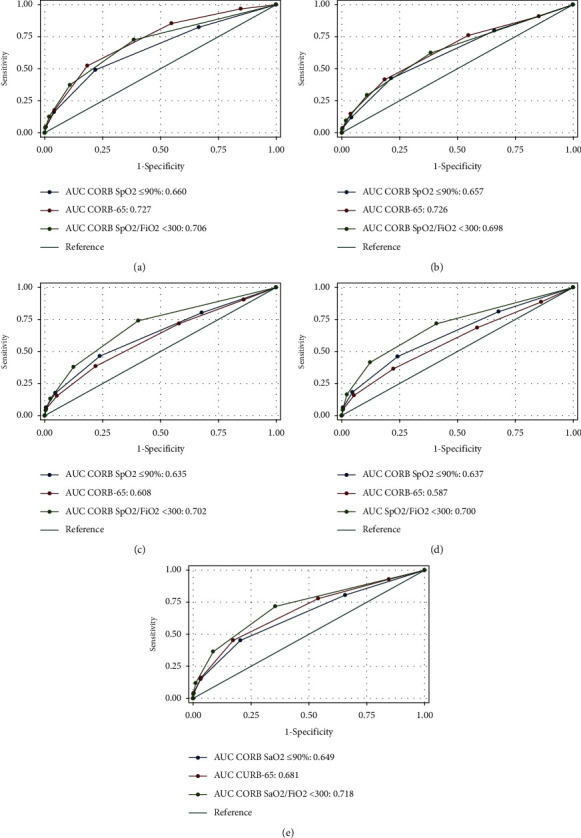
Comparison of performance between CORB score, CORB score with SpO_2_/FiO_2_ and CURB-65 (values correspond to the area under the receiver operating characteristic curve-AUC) estimated in Table 3. (a) In-hospital mortality. (b) 30-day mortality. (c) Vasopressor requirement. (d) Mechanical ventilation requirement. (e) Composite outcome (in-hospital mortality/IMV requirement/vasopressor requirement). AUC: area under the receiver operating characteristics curve. CORB: confusion (new onset or deterioration of preexisting condition), oxygen saturation ≤90%, respiratory rate ≥30/min, and systolic blood pressure <90 mmHg or diastolic blood pressure ≤60 mmHg. CURB-65: confusion, urea nitrogen >7 mmol/L (19 mg/dL), respiratory rate ≥30/min, systolic blood pressure <90 mmHg or diastolic blood pressure ≤60 mmHg, and age ≥65 years. FiO2: fraction of inspired oxygen. SpO_2_: oxygen saturation by pulse oximetry. SpO_2_/FiO_2_: oxygen saturation by pulse oximetry/fraction of inspired oxygen ratio.

Sensitivity for mortality, IMV, and vasopressor support outcomes was higher for CURB-65. Specificity was higher for CORB with SpO_2_ ≤90% for in-hospital and 30-day mortality outcomes; however, when the CORB is used with SpO_2_/FiO_2_ <300 in its score, specificity improves for vasopressor support and IMV outcomes. In composite results, high sensitivity was found in the CORB ≥2 (SpO_2_/FiO_2_ <300) with 91.3% and specificity in the CURB-65 > 2 with 77.9%.  Table 3 and Figure 2 show the complete performance results for each of the CORB scores (with SpO_2_ ≤90% and with SpO_2_/FiO_2_ <300) and CURB-65 regarding in-hospital mortality, 30-day mortality, and requirement for vasopressor support and IMV outcomes and composite outcomes.

## 4. Discussion

It was found that the CORB score presents a good performance as a predictor of IMV and vasopressor support requirement, being superior to CURB-65 in estimating these outcomes. In the evaluation of in-hospital and 30-day mortality, CURB-65 shows higher performance than CORB calculated with SpO_2_ ≤90% and CORB calculated with SpO_2_/FiO_2_ <300. On the other hand, replacing the oxygenation parameter of SpO_2_ ≤90% of the CORB score with the SpO_2_/FiO_2_ <300 index turns out to be superior in the prediction of outcomes in pneumonia.

The inclusion of oxygenation parameters in the CORB score improves the performance for the prediction of IMV compared to the CURB-65. In addition, it is necessary to clarify that the CURB-65 was designed to predict only 30-day mortality, not other outcomes such as the need for IMV or vasopressors [8]. The measurement of oxygen saturation, through pulse oximetry or by calculating the SpO_2_/FiO_2_ index, has been correlated with different degrees of hypoxemia in the patients with pneumonia [14, 15]. In the context of acute respiratory failure and adult respiratory distress syndrome (ARDS), PaO_2_/FiO_2_ represents one of the most important physiological variables for determining the degree of lung injury [16], and it is also a severity benchmark for defining severe pneumonia [5]. In an observational study carried out by Luna et al. which included 63 patients with ventilator-associated pneumonia, it was found that PaO_2_/FiO_2_ was the factor with the greatest discrimination to predict short-term survival [17]. Regarding the particular case of pneumonia at high altitude, Martínez et al. argued that the measurement of oxygenation through D(A-a)O_2_ and PaO^2^/FiO^2^ of arterial gases showed the best discrimination capacity for IMV at 2,600 meters above sea level [18].

In a multivariate analysis, Buising et al. found that the urea level and age ≥65 years proposed in CURB-65 were not strongly associated variables for the composite outcome of death, IMV, or vasopressor support (OR = 1.71; 95% CI: 0.82–3,58; OR = 0.52; 95% CI: 0,23–1,16, respectively), so the authors proposed to eliminate age and replace urea by SpO_2_, which improved the association for prediction of severity by CAP (OR = 3,49; 95% CI: 1,77–6,89) [10], results that are similar to those reported in our study where oxygenation levels were more associated with the requirement for IMV and vasopressor support. Removing the variable associated with age ≥65 years can reduce the misperception that some scores may present with the mortality outcome, decreases the possibility of obtaining higher scores for patients with advanced age who are not comorbid, and reduces the proportion of low scores in younger patients with severe CAP pictures when incorporating a variable correlated with different degrees of hypoxemia. Moreover, excluding the age variable may favor the use of the CORB score in the daily evolution of the patient, since this score would be entirely composed of variables that can change rapidly.

Babu et al. analyzed if the noninvasive index SpO_2_/FiO_2_ ratio could replace invasive index PaO_2_/FiO_2_ in all modes of oxygen supplementation in acute hypoxemic respiratory failure; a total of 300 patients were included in this study [19]. In the result, a strong positive linear correlation was noted between PF ratio and SF ratio (*r* = 0,66; *p* < 0.001). Moreover, SF values are 285 and 323 corresponding to PF ratios of 200 and 300 with a sensitivity and specificity of 70 to 80%, respectively. Similar data were found in the study carried out by Pandharipande et al. calculating the respiratory parameter of the SOFA score with noninvasive index SpO_2_/FiO_2_ and index PaO_2_/FiO_2_ with a strong positive correlation [20]. Currently, SpO_2_/FiO_2_ is a diagnostic tool for the early recognition of respiratory failure and the need for IVM, because the pulse oximeter is simple, inexpensive, and available for continuous monitoring of saturation.

Replacing SpO_2_ <90% with SpO_2_/FiO_2_ <300 ratio in CORB score significantly improves the AUC-ROC for pneumonia outcomes. Some authors suggest assessing oxygenation status through indices including FiO_2_ since they have shown good prediction of mortality in ARDS [21]. The persistent decrease of PaO_2_/FiO_2_ is related to significant alveolar involvement in patients with pneumonia as well as to the progression to ARDS; PaO_2_/FiO_2_ values less than 150 have been associated with a worse prognosis [22]. Arterial blood gas measurement is the gold standard for oxygenation assessment; however, it is an invasive, time-consuming, and uncomfortable procedure for patients. Considering these difficulties, the use of indices from pulse oximetry may be a valid alternative; SpO_2_/FiO_2_ has shown a reliable correlation with the PaO_2_/FiO_2_ index [23]. A SpO_2_/FiO_2_ ratio of 235–315 is equivalent to PaO_2_/FiO_2_ levels of 200–300 [23]; the SpO_2_/FiO_2_ <300 would be equivalent to moderate or severe hypoxemia, a parameter that is considered a minor criterion for pneumonia according to ATS/IDSA [5, 7].

This study has several limitations. It is a single-center study, which could restrain its generalizability, although the good sample size supports the results. In addition, subjects older than 18 years were enrolled, which restricts its applicability in younger groups, but adult population included is considered representative. Moreover, since it is a retrospective study, the quality of the information may be affected in the completion of the medical records, but the reduction of the transcription bias could be achieved by the verification carried out by at least two study investigators in charge of corroborating the data obtained from the clinical records. Furthermore, an altitude of 2,640 m.a.s.l. at which the cohort was evaluated could be considered a limitation to the generalization of the results due to acclimatization phenomena [24, 25]. However, there are no significant variations in the results compared to previous studies carried out at sea level [10]. Prospective studies are required to corroborate the performance of the CORB scale with the modification of the oxygenation parameter.

## 5. Conclusions

The CORB scale for pneumonia proves to have a good performance for the prediction of IMV and vasopressor support, with a higher performance when the SpO_2_/FiO_2_ ratio <300 is used as an oxygenation parameter. Finally, when compared to the CURB-65 scale, the CORB scale is not superior in predicting mortality.

## Figures and Tables

**Table 1:** General characteristics of the population.

Variable	Total population (*n* = 1,811)	Dead (*n* = 273)	Alive (*n* = 1,538)	*p* value
Age in years, x (SD)	72.89 (17.09)	80.32 (13.21)	71.57 (17.36)	<0.001
Male gender, *n* (%)	1025 (56.6)	154 (56.4)	871 (56.6)	0.946
Background, *n* (%)				
High blood pressure	1142 (63.1)	198 (72.5)	944 (61.4)	<0.001
Chronic heart failure	394 (21.8)	86 (31.5)	308 (20.0)	<0.001
DM	268 (14.8)	47 (17.2)	221 (14.4)	0.222
Dementia	304 (16.8)	95 (34.8)	209 (13.6)	<0.001
Chest X-ray, *n* (%)				
Alveolar infiltrates	1391 (76.8)	235 (86.1)	1156(75.2)	<0.001
Multi-lobar impairment	545 (30.1)	153 (56.0)	392 (25.5)	<0.001
Chest CT, *n* (%)				
Alveolar infiltrates	542 (84.4)	66 (90.4)	476 (83.7)	0.134
Multi-lobar impairment	318 (49.5)	56 (76.7)	262 (46.0)	<0.001

x (SD): average (standard deviation). n (%): number (percentage). DM: diabetes mellitus. CT: computerized tomography.

**Table 2:** Association of the CORB, CURB-65, and SpO_2_/FiO_2_ variables with each outcome.

Outcome	OR	CI 95% (inf-sup)	*p* value
In-hospital mortality			
CURB-65 ≥ 2	4,9	3.707–6.364	<0.001
CORB ≥2	3,4	2.632–4.484	<0.001
CORB ≥2 (SpO_2_/FiO_2_ <300)	4,9	3.629–6.52	<0.001
30-day mortality			
CURB-65 ≥ 2	1,4	1.266–1.46	<0.001
CORB ≥2	1,3	1.185–1.342	<0.001
CORB ≥2 (SpO_2_/FiO_2_ <300)	1,5	1.316–1.602	<0.001
IMV requirement			
CURB-65 ≥ 2	2,0	1.417–2.814	<0.001
CORB ≥2	2,7	1.92–3.726	<0.001
CORB ≥2 (SpO_2_/FiO_2_ <300)	5,0	3.557–7.133	<0.001
Vasopressor requirement			
CURB-65 ≥ 2	2,2	1.601–3.066	<0.001
CORB ≥2	2,8	2.013–3.792	<0.001
CORB ≥2 (SpO_2_/FiO_2_ <300)	4,3	3.064–6.017	<0.001
Composite outcome (in-hospital mortality/IMV requirement/vasopressor requirement)			
CURB-65 ≥ 2	3,9	3.103–5.008	<0.001
CORB ≥2	3,2	2.540–4.059	<0.001
CORB ≥2 (SpO_2_/FiO_2_ <300)	6,0	4.564–7.900	<0.001

OR: odds ratio. CI: confidence interval. IMV: invasive mechanical ventilation. SpO_2_/FiO_2_: oxygen saturation by pulse oximetry/fraction of inspired oxygen ratio. CURB-65: confusion, urea nitrogen >7 mmol/L (19 mg/dL), respiratory rate ≥30/min, systolic blood pressure <90 mmHg or diastolic blood pressure ≤60 mmHg, and age ≥65 years. CORB: confusion (new onset or deterioration of preexisting condition), oxygen saturation ≤90%, respiratory rate ≥30/min, and systolic blood pressure <90 mmHg or diastolic blood pressure ≤60 mmHg. Hosmer–Lemeshow test: 0,598; 0,238; 0,498; 0,247; 0,328.

**Table 3:** Performance of CORB and CURB-65 scores for mortality, vasopressor support, and IMV outcomes.

	S	E	PPV	NPV	LR+	LR−	AUC	CI 95%(inf-sup)	*p* value
In-hospital mortality									
CORB ≥2 (SpO_2_ ≤90%)	49,1%	78,1%	28,5%	89,6%	2,24	0,65	0,660	(0.623–0.697)	<0.001
CORB ≥2 (SpO_2_/FiO_2_ <300)	72,5%	61,4%	25,0%	92,6%	1,88	0,45	0,706	(0.67–0.741)	<0.001
CURB-65 ≥ 2	85,3%	45,2%	21,7%	94,6%	1,56	0,32	0,727	(0.695–0.759)	<0.001
30-day mortality									
CORB ≥2 (SpO_2_ ≤90%)	21,8%	52,5%	8,3%	77,4%	0,46	1,49	0,657	(0.621–0.692)	<0.001
CORB ≥2 (SpO_2_/FiO_2_ <300)	38,3%	28,6%	9,5%	70,3%	0,54	2,16	0,698	(0.664–0.733)	<0.001
CURB-65 ≥ 2	54,5%	14,5%	11,1%	61,8%	0,64	3,15	0,726	(0.695–0.756)	<0.001
Vasopressor support									
CORB ≥2 (SpO_2_ ≤90%)	46,3%	76,2%	25,7%	88,9%	1,95	0,70	0,635	(0.589–0.681)	<0.001
CORB ≥2 (SpO_2_/FiO_2_ <300)	37,9%	87,6%	35,1%	88,8%	3,05	0,71	0,702	(0.66–0.745)	<0.001
CURB-65 ≥ 2	71,8%	41,9%	18,0%	89,3%	1,24	0,67	0,608	(0.562–0.654)	<0.001
IMV									
CORB ≥2 (SpO_2_ ≤90%)	43,0%	76,2%	24,3%	88,3%	1,81	0,75	0,637	(0.589–0.685)	<0.001
CORB ≥2 (SpO_2_/FiO_2_ <300)	37,7%	88,0%	35,8%	88,8%	3,15	0,71	0,700	(0.654–0.746)	<0.001
CURB-65 ≥ 2	71,0%	42,1%	17,9%	89,1%	1,23	0,69	0,587	(0.538–0.637)	<0.001
Composite outcome (in-hospital mortality/IMV requirement/vasopressor requirement)									
CORB ≥2 (SpO_2_ ≤90%)	45,2%	42,1%	28,2%	89,1%	2,21	0,69	0,64	(0,618–0,681))	0,016
CORB ≥2 (SpO_2_/FiO_2_ <300)	36,4%	91,3%	42,6%	89,0%	4,18	0,70	0,71	(0,688–0,748)	0,015
CURB-65 ≥ 2	77,9%	45,9%	20,4%	92,1%	1,44	0,48	0,68	(0,651–0,712)	0,016

S: sensibility. E: specificity. PPV: positive predictive value. NPV: negative predictive value. LR+: positive likelihood ratio. LR−: negative likelihood ratio. AUC: area under the curve of receiver operating characteristics. CI: confidence interval. CORB: confusion (new onset or deterioration of preexisting condition), oxygen saturation ≤90%, respiratory rate ≥30/min, and systolic blood pressure <90 mmHg or diastolic blood pressure ≤60 mmHg. CURB-65: confusion, urea nitrogen >7 mmol/L (19 mg/dL), respiratory rate ≥30/min, systolic blood pressure <90 mmHg or diastolic blood pressure ≤60 mmHg, and age ≥65 years. FiO_2_: fraction of inspired oxygen. SpO_2_: oxygen saturation by pulse oximetry. SpO_2_/FiO_2_: oxygen saturation by pulse oximetry/fraction of inspired oxygen ratio.

## Data Availability

Data supporting the findings of this study are in medical records from the research center.

## Abbreviations

AUC: Area under the curve of receiver operating characteristics
CORB: Confusion (new onset or deterioration of preexisting condition), oxygen saturation ≤90%, respiratory rate ≥30/min, and systolic blood pressure <90 mmHg or diastolic blood pressure ≤60 mmHg
CURB-65: Confusion, urea nitrogen >7 mmol/L (19 mg/dL), respiratory rate ≥30/min, systolic blood pressure <90 mmHg or diastolic blood pressure ≤60 mmHg, and age ≥65 years
MSL: Meters above sea level
CAP: Community-acquired pneumonia
SpO_2_: Oxygen saturation by pulse oximetry
SpO_2_/FiO_2_: Oxygen saturation by pulse oximetry/fraction of inspired oxygen ratio
ICU: Intensive care unit
IMV: Invasive mechanical ventilation.

## Supplementary Materials

Supplementary Materials Results with risk score variables of in-hospital mortality, 30-day mortality, invasive mechanical ventilation requirement, vasopressor requirement, and composite outcome.
