# Supplementary material for: Performance of the CORB (Confusion, Oxygenation, Respiratory Rate, and Blood Pressure) Scale for the Prediction of Clinical Outcomes in Pneumonia
Source: Can Respir J. 2022 Jun 3;2022:4493777. doi: 10.1155/2022/4493777 (PMC9187474; doi:10.1155/2022/4493777)
Supplement: Supplementary Materials — Results with risk score variables of in-hospital mortality, 30-day mortality, invasive mechanical ventilation requirement, vasopressor requirement, and composite outcome. [file 4493777.f1.docx]

**Supplementary materials**

| Supplementary file 1. Outcomes with the risk scores variables | | | | | | |
| --- | --- | --- | --- | --- | --- | --- |
| Outcome | OR | CI 95% (Inf-Sup) | p value | AOR | CI 95%(Inf-Sup) | p value |
| In-hospital mortality |  |  |  |  |  |  |
| Confusion | 4,1 | 3.114-5.527 | <0.001 | 2,4 | 1.782-3.334 | <0.001 |
| BUN >19 mg/dl | 3,2 | 2.388-4.328 | <0.001 | 2,2 | 1.596-3.007 | <0.001 |
| RR ≥ 30 rpm | 3,0 | 2.006-4.438 | <0.001 | 1,7 | 1.089-2.692 | 0.02 |
| SBP <90 mmHg or DBP ≤ 60 mmHg | 2,2 | 1.654-2.86 | <0.001 | 1,6 | 1.22-2.215 | 0.001 |
| Age ≥65 years | 3,3 | 2.225-5.018 | <0.001 | 2,3 | 1.464-3.482 | <0.001 |
| SpO_2_ ≤ 90% | 1,4 | 1.096-1.858 | 0.008 | - | - | 0.321 |
| SpO2/FiO_2_<300 | 4,2 | 3.136-5.622 | <0.001 | 3,0 | 2.192-4.144 | <0.001 |
| 30 days mortality |  |  |  |  |  |  |
| Confusion | 1,4 | 1.262-1.505 | <0.001 | 2,4 | 1.761-3.247 | <0.001 |
| BUN >19 mg/dl | 1,2 | 1.146-1.242 | <0.001 | 2,3 | 1.661-3.06 | <0.001 |
| RR ≥ 30 rpm | 1,3 | 1.132-1.456 | <0.001 | 1,7 | 1.093-2.663 | 0.019 |
| SBP <90 mmHg or DBP ≤ 60 mmHg | 1,2 | 1.088-1.224 | <0.001 | 1,6 | 1.207-2.155 | 0.001 |
| Age >65 years | 1,2 | 1.129-1.212 | <0.001 | 2,6 | 1.664-3.927 | <0.001 |
| SpO_2_ ≤ 90% | 1,1 | 1.016-1.101 | 0.007 | - | - | 0.322 |
| SpO_2_/FiO_2_ <300 | 1,4 | 1.249-1.496 | <0.001 | 2,8 | 2.035-3.812 | <0.001 |
| IMV requirement |  |  |  |  |  |  |
| Confusion | 3,1 | 2.142-4.348 | <0.001 | 2,2 | 1.439-3.312 | <0.001 |
| BUN >19 mg/dl | 1,7 | 1.18-2.332 | 0.003 | 1,6 | 1.072-2.347 | 0.021 |
| RR ≥ 30 rpm | 4,8 | 3.113-7.358 | <0.001 | 2,6 | 1.562-4.2 | <0.001 |
| SBP <90 mmHg or DBP ≤ 60 mmHg | 1,3 | 0.927-1.91 | 0.12 | - | - | 0.86 |
| Age ≥65 years | 0,5 | 0.389-0.77 | <0.001 | 0,3 | 0.213-0.479 | <0.001 |
| SpO_2_ ≤ 90% | 1,7 | 1.203-2.384 | 0.002 | 1,5 | 1.017-2.132 | 0.04 |
| SpO_2_/FiO_2_ <300 | 6,7 | 4.778-9.509 | <0.001 | 5,1 | 3.525-7.481 | <0.001 |
| Vasopressor requirement |  |  |  |  |  |  |
| Confusion | 2,8 | 2-3.967 | <0.001 | 2,0 | 1.327-2.91 | 0.001 |
| BUN >19 mg/dl | 1,7 | 1.213-2.322 | 0.002 | 1,6 | 1.085-2.249 | 0.016 |
| RR ≥ 30 rpm | 4,1 | 2.699-6.322 | <0.001 | 2,5 | 1.542-3.964 | <0.001 |
| SBP <90 mmHg or DBP ≤ 60 mmHg | 1,9 | 1.399-2.695 | <0.001 | 1,6 | 1.11-2.242 | 0.011 |
| Age ≥65 years | 0,6 | 0.456-0.886 | 0.007 | 0,4 | 0.283-0.604 | <0.001 |
| SpO_2_ ≤ 90% | 1,4 | 1.002-1.892 | 0.048 | - | - | 0.411 |
| SpO_2_/FiO_2_ <300 | 4,6 | 3.288-6.413 | <0.001 | 3,5 | 2.401-4.959 | <0.001 |
| Composite outcome (In-hospital mortality/ IMV requirement / Vasopressor requirement) | | | | | | |
| Confusion | 4,5 | (3.453-5.844) | <0.001 | 3,0 | (2.222-3.969) | <0.001 |
| BUN >19 mg/dl | 2,4 | (1.913-3.076) | <0.001 | 1,9 | (1.466-2.452) | <0.001 |
| RR ≥ 30 rpm | 4,3 | (2.950-6.156) | <0.001 | 2,6 | (1.728-4.017) | <0.001 |
| SBP <90 mmHg or DBP ≤ 60 mmHg | 1,9 | (1.514-2.457) | <0.001 | 1,5 | (1.112-1.911) | 0.006 |
| Age ≥65 years | 1,4 | (1.068-1.828) | <0.001 | 1,4 | (1.234-1.893) | 0.377 |
| SpO_2_ ≤ 90% | 1,3 | (1.008-1.576) | <0.001 | - | - | 0.993 |
| SpO_2_/FiO_2_ <300 | 6,0 | (4.570-7.862) | <0.001 | 4,4 | (3.309-5.920) | <0.001 |
| Abbreviations OR: Odds Ratio. AOR: Adjusted Odds Ratio. BUN: Blood Urea Nitrogen. RR: Respiratory Rate. SBP: Systolic Blood Pressure. DBP: Diastolic Blood Pressure. SpO2: Oxygen saturation by pulse oximetry. SpO2/FiO2: Oxygen saturation by pulse oximetry / fraction of inspired oxygen ratio. CORB: Confusion (new onset or deterioration of pre-existing condition), oxygen saturation ≤ 90%, respiratory rate ≥ 30 / min, and systolic blood pressure <90 mmHg or diastolic blood pressure ≤ 60 mmHg | | | | | | |
